# Plasma triglyceride is associated with the recurrence of atrial fibrillation after radiofrequency catheter ablation: A retrospective study

**DOI:** 10.1002/clc.24276

**Published:** 2024-05-30

**Authors:** Qingqing Gu, Ye Deng, Jun Wei, Qianwen Chen, Dabei Cai, Tingting Xiao, Li Deng, Yu Wang, Qingjie Wang, Ling Sun, Yuan Ji

**Affiliations:** ^1^ Department of Cardiology The Affiliated Changzhou Second People's Hospital of Nanjing Medical University, Changzhou Medical Center Changzhou Jiangsu China; ^2^ Department of Cardiovascular Surgery The Affiliated Hospital of Xuzhou Medical University Xuzhou Jiangsu China; ^3^ Department of Cardiovascular Surgery The First Affiliated Hospital of Wannan Medical College Wuhu Anhui China; ^4^ Dalian Medical University Dalian Liaoning China

**Keywords:** atrial fibrillation, catheter ablation, recurrence, triglycerides

## Abstract

**Background:**

The purpose of this study was to explore the association between triglycerides (TGs) and the risk of atrial fibrillation (AF) recurrence.

**Methods and Results:**

Included were adult patients with AF who underwent radiofrequency catheter ablation in the Affiliated Changzhou Second People's Hospital of Nanjing Medical University. The enrolled patients were divided into the AF recurrence group and the sinus rhythm (SR) maintenance group. The univariate Cox regression analysis and Kaplan–Meier survival curve were performed estimate the association between TG and the risk of AF recurrence. Of the 402 patients, 79 (19.7%) experienced recurrence of AF after ablation. The TG level was significantly higher in the AF recurrence group than in the SR‐maintaining group. Patients were grouped by quartile of TG levels, with Quartile 1 and Quartile 2 defined as the low concentration group, Quartile 3 as the moderate concentration group, and Quartile 4 as the high concentration group. Multivariate Cox regression analysis showed that the moderate concentration group (*p* = .02, hazard ratio [HR]: 2.331, 95% confidence interval [CI]: 1.141–4.762) and high concentration group (*p* = .007, HR: 2.873, 95% CI: 1.332–6.199) were associated with an increased risk of AF recurrence compared with the low concentration group. The median follow‐up was 1.17 years, it is indicated that a higher risk of recurrent AF was observed in the moderate concentration and high concentration group (log‐rank: *χ* ^2^ = 7.540, *p* = .023).

**Conclusion:**

Our data suggest that an elevated TG level measured before catheter ablation is associated with an increased risk of AF recurrence.

## Graphical abstract

We uncovered that the triglyceride (TG) level was significantly higher in the atrial fibrillation (AF) recurrence group than in the sinus rhythm‐maintaining group. Cox regression showed that elevated plasma TG level before catheter ablation was associated with an increased risk of AF recurrence. A higher risk of AF recurrence was observed in the moderate and high TG concentration groups.

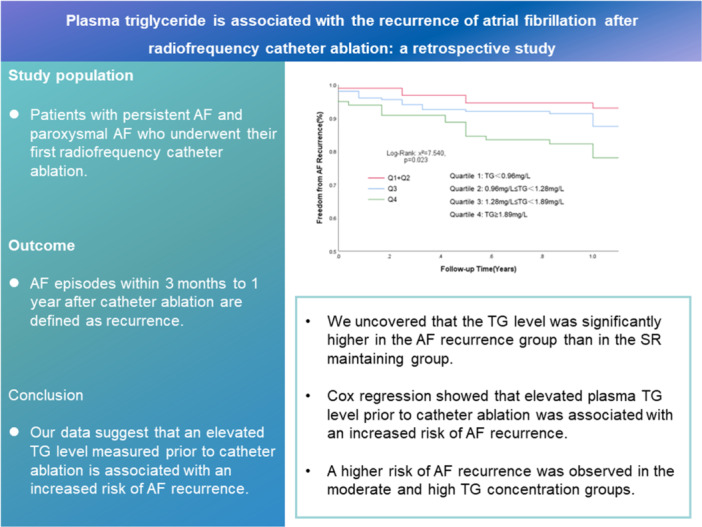

**Abbreviations**
AF: atrial fibrillation
ATGL: adipose triglyceride lipase
BMI: body mass index
HDL‐C: high‐density lipoprotein cholesterol
LDL‐C: low‐density lipoprotein cholesterol
SR: sinus rhythm
TCH: total cholesterol
TG: triglycerides
TSH: thyroid‐stimulating hormone

## 1 INTRODUCTION

Atrial fibrillation (AF), the most common arrhythmia in clinical practice, brings with increased risk of stroke, heart failure, and other heart‐related complications. In 2017, approximately 304.6 million new cases of AF were confirmed worldwide.^1^ Current guidelines recommend catheter ablation to improve the quality of life in patients with symptomatic and antiarrhythmic drug‐refractory AF.^2^ However, recurrence of arrhythmia after catheter ablation is common, with a rate of about 26.4% at 12 months and up to 30% beyond 12 months after catheter ablation.^3^, ^4^

Many mechanisms underlie the recurrence of AF, including lipid metabolism. Some studies report that the risk of AF is associated with high‐density lipoprotein cholesterol (HDL‐C) and triglyceride (TG), but not with low‐density lipoprotein cholesterol (LDL‐C) and total cholesterol (TCH).^5^, ^6^, ^7^ However, others reported no correlation between TG levels and the risk of AF.^8^, ^9^ This controversy may arise from bias in sample sizes. TGs, components of triglycerides‐rich lipoproteins, are insoluble in water but soluble in the blood after binding to apolipoproteins. Elevated TG level is a risk factor for cardiovascular disease.^10^ Some studies have shown that lipoprotein has a certain relationship with the occurrence, development and recurrence of AF.^11^ TG level is also positively correlated with the risk of developing AF.^12^, ^13^, ^14^, ^15^ We suspect that plasma TG level is also associated with early recurrence of AF. Our study aimed to investigate the effect of plasma TG level on AF recurrence after catheter ablation.

## 2 SUBJECTS AND METHODS

### 2.1 Study population

Included were adult patients with persistent AF and paroxysmal AF who underwent their first radiofrequency catheter ablation in the Affiliated Changzhou Second People's Hospital of Nanjing Medical University from April 29, 2018 to June 7, 2021. The study was approved by the Ethics Committee of Changzhou Second People's Hospital (No. KY325‐01), and was performed in accordance with the Helsinki Declaration. Written informed consent was obtained from all patients. Inclusion criteria: adults aged 18–80 years; patients examined with 12‐lead electrocardiogram (ECG) or Holter monitoring for AF; patients with persistent and paroxysmal AF; patients having not taken class I or III antiarrhythmic drugs; patients with symptomatic persistent AF who showed ineffectiveness or intolerance to antiarrhythmic drugs; Exclusion criteria: left atrial anteroposterior diameter >60 mm, left atrial appendage thrombosis, structural cardiac disorders (i.e., severe aorta, tricuspid or mitral valve malformations, tetralogy of Fallot, ventricular and atrial septal defects), psychiatric disorders, septic shock, glomerular filtration rate <30 mL/min, abnormal thyroid function, and severe pericardial effusion or tamponade.

### 2.2 Medical characteristic data collection

**Figure 1:**
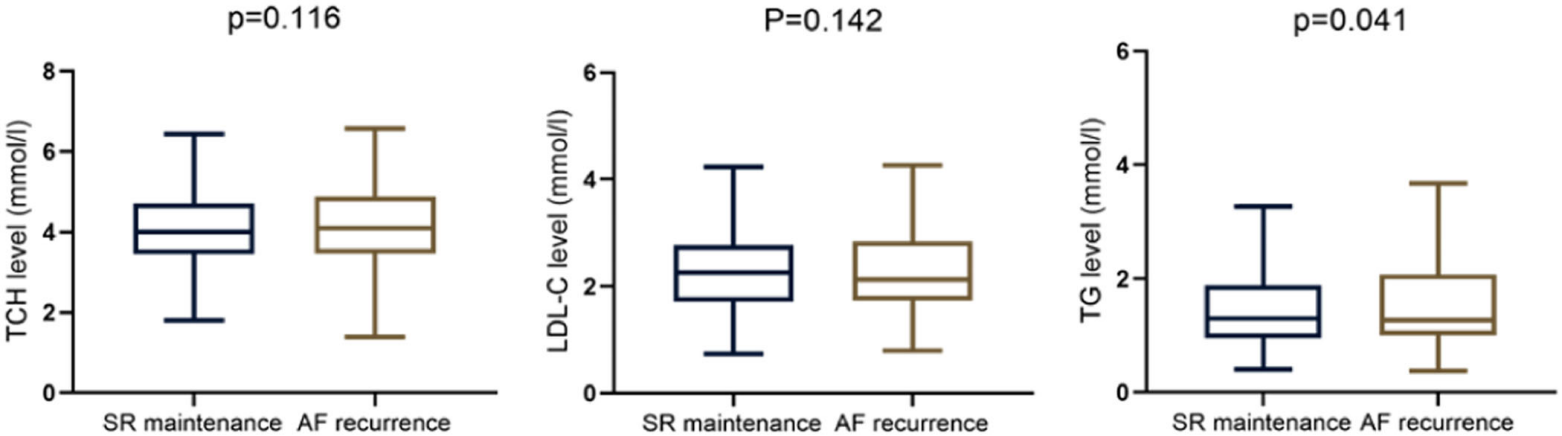
Comparison of TCH, LDL‐C, and TG levels in two groups. Box plots represent median levels with 25th and 75th percentiles of the value of variables. AF, atrial fibrillation; LDL‐C, low‐density lipoprotein cholesterol; SR, sinus rhythm; TCH, total cholesterol; TG triglycerides.

Before catheter ablation, plasma was sampled for laboratory tests. Standardized case report forms were used to collect personal and medical characteristic data at baseline, including information about demographics, lifestyle habits, medical history, and current medications. According to current guidelines, AF is divided into paroxysmal (spontaneous termination of AF within 48 h) or persistent (lasting more than 7 days, including AF terminated by cardioversion after 7 days or more). The study flowchart is illustrated in Supporting Information S1: Figure 1.

### 2.3 Catheter ablation

For catheter ablation, an electrode catheter was inserted into the heart by puncturing through the femoral vein, femoral artery, or subclavian vein under X‐ray angiography. The site of the abnormal structure causing tachycardia was located and then ablated by a high‐frequency current of 100 kHz, 5 MHz that generated a high temperature in a small range. Ablation strategies, including galvanic pulmonary venous isolation, tricuspid isthmus ablation, and left atrial linear ablation, were determined for each patient at the discretion of the surgeon.

### 2.4 Follow‐up and outcome

Patients were followed up for at least 12 months by physical examination, 24‐h Holter, or 48‐h Holter in the outpatient clinic. The recurrence of AF or atrial tachycardia lasting more than 30 s was defined as the primary study endpoint. AF episodes within 3 months to 1 year after catheter ablation are defined as recurrence.^3^ Each patient received monthly ECG for 6 months after surgery, and telephone interview every month for reporting discomfort after 1 year.

### 2.5 Statistical analysis

The statistical analysis was performed using IBM SPSS® version 26 software. The two‐sided *p* < .05 was considered statistically significant. Continuous variables in a normal distribution were reported as mean ± standard deviation, and those in nonnormal distribution as median with interquartile deviation. Categorical variables were expressed as frequency plus percentage.

The 402 cases were divided into AF recurrence group and sinus rhythm (SR) maintenance group. Continuous variables were compared using the *t* test, and categorical variables using the *χ* ^2^ test. The univariate Cox regression analysis was performed. The factors with *p* < .1 continued to be included into the multivariate regression analysis. The Kaplan–Meier survival curve was used to estimate the cumulative incidences of recurrence of AF in different quartiles of TG levels, which were then compared using a log‐rank test. We constructed a Cox proportional hazards model to assess the risk of AF recurrence at median TG levels and quartiles. The first model was not adjusted, the second model adjusted for age and sex, and the third model adjusted for age, sex, body mass index (BMI), platelet level, history of hypertension (yes vs. no), history of heart failure (yes vs. no), and type of AF.

## 3 RESULTS

From April 29, 2018 to June 7, 2021, a total of 450 subjects with AF were recruited. All patients completed at least one follow‐up visit for at least 12 months. Forty‐eight patients were lost to follow‐up at 12 months of follow‐up, leaving them out of the analysis. Of the 402 patients, 79 (19.7%) experienced recurrence of AF after ablation. AF episodes within 3 months to 1 year after catheter ablation were defined as recurrence. Patients were divided into the AF recurrence and the SR maintenance groups. Compared with the SR maintenance group, the AF recurrence group showed a significant increase in red blood cell count, TG level, and thyroid‐stimulating hormone (TSH) level, while the white blood cell count and free thyroxine level were significantly reduced (Table 1). As shown in Table 1, there was no significant difference in catheter ablation‐related indicators between the SR maintenance group and the AF recurrence group. In addition, we selected TCH, LDL‐C, and TG from the lipid index as the objects for box plot analysis. Compared with the SR maintenance group, the TG level in the AF recurrence group was significantly increased, while there was no significant difference in TCH and LDL‐C levels (Figure 1).

**Table 1:** Baseline information of SR maintenance patients and AF recurrence patients.

Variables	All (*n* = 402)	SR maintenance (*n* = 323)	AF recurrence (*n* = 79)	*p*
Age (year)	63.8 ± 9.7	64.42 ± 9.56	60.99 ± 10.04	.636
Male, *n* (%)	241 (59.8%)	193 (59.8%)	47 (59.5%)	.966
Persistent AF, *n* (%)	143 (35.5%)	119 (36.8%)	24 (30.4%)	.282
BMI (kg/m^2^)	25.06 ± 3.03	24.93 ± 3.05	25.60 ± 2.88	.536
WBC (×10^9^/L)	6.43 ± 1.80	6.44 ± 1.72	6.39 ± 2.08	.037
NEUT	61.50 ± 10.27	61.48 ± 10.32	61.61 ± 10.09	.605
RBC (×10^12^/L)	4.86 ± 6.35	4.55 ± 0.55	6.14 ± 14.33	.046
Hb (g/L)	139.71 ± 18.16	140.22 ± 17.42	137.88 ± 20.99	.188
PLT (×10^9^/L)	195.33 ± 52.25	197.82 ± 51.21	184.65 ± 55.22	.248
RDW‐CV (%)	13.07 ± 2.31	13.00 ± 2.06	13.33 ± 3.13	.213
PT (s)	15.08 ± 14.42	15.18 ± 15.81	14.67 ± 5.6	.415
INR	1.32 ± 1.31	1.33 ± 1.44	1.29 ± 0.48	.467
APTT (s)	31.68 ± 7.67	31.55 ± 7.71	32.24 ± 7.51	.619
BUN (mmol/L)	7.35 ± 20.97	7.72 ± 23.33	5.77 ± 1.40	.230
Cr (µmol/L)	75.68 ± 17.43	75.14 ± 17.42	78.01 ± 17.30	.835
UA (µmol/L)	334.77 ± 88.85	336.92 ± 90.41	326.23 ± 81.57	.183
TCH (mmol/L)	4.11 ± 0.96	4.09 ± 0.94	4.21 ± 1.05	.116
TG (mmol/L)	1.55 ± 0.96	1.52 ± 0.91	1.68 ± 1.15	.041
LDL (mmol/L)	2.28 ± 0.74	2.26 ± 0.72	2.35 ± 0.81	.142
TSH (µIU/L)	2.47 ± 2.41	2.39 ± 1.97	2.81 ± 3.70	.010
FT3 (pmol/L)	4.77 ± 2.22	4.76 ± 2.19	4.83 ± 2.35	.280
FT4 (pmol/L)	16.71 ± 3.79	16.74 ± 3.54	16.56 ± 4.71	.007
LVEF (%)	58.78 ± 5.82	58.75 ± 5.67	58.90 ± 6.49	.843
LAD (mm)	42.38 ± 5.41	42.14 ± 5.29	43.42 ± 5.89	.064
Hypertension, *n* (%)	267 (66.4%)	209 (64.7%)	58 (73.4%)	.142
Diabetes, *n* (%)	80 (19.9%)	62 (19.2%)	18 (22.8%)	.474
Symptomatic heart failure, *n* (%)	57 (14.2%)	47 (14.6%)	10 (12.7%)	.666
CHA_2_DS_2_‐VASc score	2.53 ± 1.07	2.51 ± 1.05	2.58 ± 1.18	.245
Duration of ablation (h)	3.03 ± 0.98	3.04 ± 0.98	3.01 ± 0.94	.540
Heparin total dose (IU)	7855.10 ± 2480.79	7739.94 ± 2474.19	8325.95 ± 2467.53	.999
RFCA strategy				
PVI only, *n* (%)	189 (47%)	153 (47.4%)	36 (45.6%)	.824
PVI + CTI ablation, *n* (%)	85 (21.1%)	70 (21.7%)	15 (19.0%)	
PVI+ left atrial linear ablation, *n* (%)	116 (28.9%)	92 (28.5%)	24 (30.4%)	

*Note*: Data are presented as means ± standard deviations or medians (interquartile range), and counts (percentages).

Abbreviations: AF, atrial fibrillation; APTT, activated partial thromboplastin time; BMI, body mass index; BUN, blood urea nitrogen; Cr, creatinine; CTI, cavotricuspid isthmus; FT3, free triiodothyronine; FT4, free thyroxine; Hb, hemoglobin; INR, international normalized ratio; LAD, left atrial diameter; LDL, low‐density lipoprotein; LVEF left ventricular ejection fraction; NEUT, neutrophil count; PLT, blood platelet; PT, prothrombin time; PVI pulmonary venous isolation; RBC, red blood cell; TCH, total cholesterol; TG, triglycerides; TSH, thyroid‐stimulating hormone; WBC, white blood cell; RDW‐CV, red cell volume distribution width; RFCA, radiofrequency ablation of cardiac catheter; SR, sinus rhythm; UA uric acid.

In the univariate analysis, only BMI, platelet count, TG level, and TSH level were significantly associated with AF recurrence (Table 2). Include the above variables in a multivariate analysis. The association of platelet count (*p* = .046, hazard ratio [HR]: 0.995, 95% confidence interval [CI]: 0.991–1.000) and TG level (*p* = .033, HR: 1.251, 95% CI: 1.019–1.536) with the recurrence of AF remained significant in the multivariate analysis. AF recurrence was not associated with BMI or TSH level (*p* = .166, HR: 1.053, 95% CI: 0.979–1.134; *p* = .054, HR: 1.084, 95% CI: 0.999–1.176, respectively).

**Table 2:** Univariate and multivariate Cox proportional hazards regression analysis of AF recurrence.

Variables	Univariate		Multivariable	
	Hazard ratio (95% CI)	*p*	Hazard ratio (95% CI)	*p*
Age (year)	0.985 (0.964–1.006)	.165		
Male, *n* (%)	1.125 (0.715–1.770)	.611		
Persistent AF, *n* (%)	1.112 (0.686–1.803)	.666		
BMI (kg/m^2^)	1.071 (0.997–1.149)	.059	1.053 (0.979–1.134)	.166
WBC (×10^9^/L)	0.987 (0.876–1.112)	.833		
NEUT (%)	1.007 (0.986–1.029)	.530		
RBC (×10^12^/L)	1.012 (0.996–1.028)	.156		
Hb (g/L)	0.996 (0.985–1.007)	.445		
PLT (×10^9^/L)	0.996 (0.991–1.000)	.054	0.995 (0.991–1.000)	.046
RDW‐CV (%)	1.057 (0.988–1.130)	.107		
PT (s)	0.992 (0.996–1.018)	.535		
INR	0.931 (0.692–1.252)	.637		
APTT (s)	1.002 (0.975–1.030)	.893		
BUN (mmol/L)	0.989 (0.946–1.033)	.620		
Cr (µmol/L)	1.006 (0.994–1.019)	.320		
UA (µmol/L)	1.000 (0.997–1.002)	.659		
TCH (mmol/L)	1.080 (0.850–1.373)	.527		
TG (mmol/L)	1.251 (1.030–1.519)	.024	1.251 (1.019–1.536)	.033
LDL (mmol/L)	1.100 (0.807–1.498)	.547		
TSH (µIU/L)	1.087 (1.003–1.179)	.069	1.084 (0.999–1.176)	.054
FT3 (pmol/L)	1.019 (0.929–1.119)	.687		
FT4 (pmol/L)	1.001 (0.941–1.064)	.983		
LVEF (%)	1.005 (0.965‐1.047)	.808		
LAD (mm)	1.058 (0.997–1.082)	.101		
Hypertension, *n* (%)	0.680 (0.409–1.131)	.137		
Diabetes, *n* (%)	1.210 (0.713–2.054)	.479		
Symptomatic heart failure, *n* (%)	0.799 (0.397–1.609)	.530		
CHA_2_DS_2_‐VASc score	1.053 (0.868–1.276)	.601		
Duration of ablation (h)	0.949 (0.755–1.194)	.657		
Heparin total dose (IU)	1.032 (0.874–1.199)	.451		
PVI only, *n* (%)	1.377 (0.330–5.752)	.935		

Abbreviations: AF, atrial fibrillation; APTT, activated partial thromboplastin time; BMI, body mass index; BUN, blood urea nitrogen; CI, confidence interval; Cr, creatinine; FT3, free triiodothyronine; FT4, free thyroxine; Hb, hemoglobin; INR, international normalized ratio; LAD, left atrial diameter; LDL, low‐density lipoprotein; LVEF, left ventricular ejection fraction; NEUT, neutrophil count; PLT, blood platelet; PT, prothrombin time; PVI, pulmonary venous isolation; RBC, red blood cell; TCH, total cholesterol; TG, triglycerides; TSH, thyroid‐stimulating hormone; WBC, white blood cell; RDW‐CV, red cell volume distribution width; UA uric acid.

Patients were then grouped by quartile of TG levels, with Quartile 1 and Quartile 2 defined as the low concentration group, Quartile 3 as the moderate concentration group, and Quartile 4 as the high concentration group. In the univariate Cox regression analysis, the risk of AF recurrence is higher in the moderate (*p* = .034, HR: 2.111, 95% CI: 1.056–4.218) and high (*p* = .008, HR: 2.834, 95% CI: 1.316–6.105) concentration groups than in the low concentration group. When adjusted for age and gender, the results are the same as before. Finally, adjustments were made based on age, gender, BMI, platelet levels, history of hypertension, history of heart failure, and AF type, the multivariate Cox regression analysis showed that the moderate concentration group (*p* = .02, HR: 2.331, 95% CI: 1.141–4.762) and the high concentration group (*p* = .007, HR: 2.873, 95% CI: 1.332–6.199) were associated with an increased risk of AF recurrence compared with the low concentration group (Table 3).

**Table 3:** Hazard ratios for recurrent AF after catheter ablation according to TG quartiles and continuous TG levels.

Variables		Quartile		
		Q1 + Q2	Q3	Q4
Median and quartile value, mmol/L		<1.28	1.28–1.895	>1.895
Model 1	Hazard ratio unadjusted	1	2.111	2.834
	95% CI	–	1.056–4.218	1.316–6.105
	*p* Value	–	.034	.008
Model 2	Hazard ratio adjusted for BMI	1	2.421	2.864
	95% CI	–	1.191–4.923	1.328–6.117
	*p* Value	–	.015	.007
Model 3	Hazard ratio adjusted for BMI+ additional factors	1	2.331	2.873
	95% CI	–	1.141–4.762	1.332–6.199
	*p* Value	–	.020	.007

*Note*: Model 1 was not adjusted, Model 2 adjusted for age and sex, and Model 3 adjusted for age, sex, body mass index, platelet level, history of hypertension, history of heart failure, and type of AF.

According to the TG quartile, it was divided into four groups, Quartile 1 and Quartile 2 defined as the low concentration group (TG < 1.28 mg/L), Quartile 3 as the moderate concentration group (1.28 ≤ TG < 1.89 mg/L), and Quartile 4 as the high concentration group (TG ≥ 1.89 mg/L).

Abbreviations: AF, atrial fibrillation; BMI, body mass index; TG, triglycerides.

**Figure 2:**
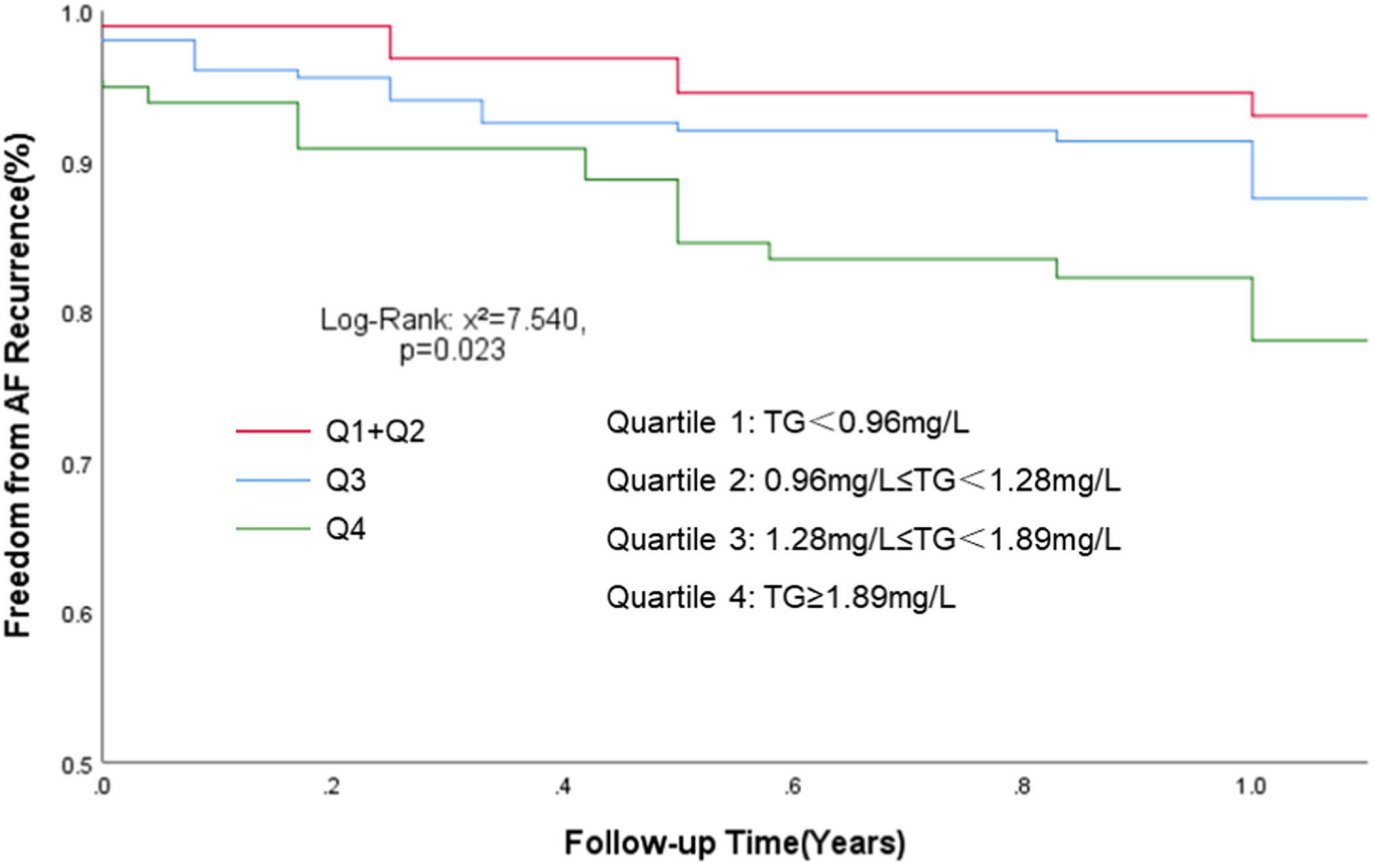
Kaplan–Meier curves for AF recurrence across quartile of TG. It was divided into four groups, Quartile 1 and Quartile 2 defined as the low concentration group (TG < 1.28 mg/L), Quartile 3 as the moderate concentration group (1.28 ≤ TG < 1.89 mg/L), and Quartile 4 as the high concentration group (TG ≥ 1.89 mg/L). AF, atrial fibrillation; TG, triglyceride.

The median follow‐up was 1.17 years, Kaplan–Meier survival curve showed a higher risk of recurrence of AF was observed in the moderate concentration and high concentration groups (log‐rank: *χ* ^2^ = 7.540, *p* = .023) (Figure 2).

## 4 DISCUSSION

The main findings of this study are as follows: (1) In this retrospective analysis of patients with AF undergoing catheter ablation, we for the first time uncovered that the TG level was significantly higher in the AF recurrence group than in the SR‐maintaining group. (2) Subsequently, Cox regression showed that elevated plasma TG level before catheter ablation was associated with an increased risk of AF recurrence. And after a wide range of potential confounding factors were adjusted, it also indicated that the highest TG level group had a 2.873‐fold higher risk of AF recurrence than the lowest TG group. (3) Finally, a higher risk of AF recurrence was observed in the moderate and high TG concentration groups. These results suggest that a good control of plasma TG levels in AF patients before catheter ablation may reduce the risk of postoperative recurrence of AF.

The recurrence of AF after ablation therapy may be associated with several mechanisms, including lipid metabolism. Recent studies have found that lipoprotein(a) (Lp(a)), HDL‐c, VLDL‐c, and TG are metabolized together at high TG levels.^16^, ^17^ In addition, postprandial lipoprotein(a) elevates with the levels of plasma TGs,^18^ suggesting that TGs are closely related to lipoprotein(a). Recent research has found that Lp(a) is involved in the occurrence and development of coronary heart disease.^19^, ^20^, ^21^ Lp(a) has also been found as a potential causal mediator in the development of AF.^22^ A recent large retrospective cohort study has shown that circulating Lp(a) level is inversely correlated with the risk of AF development.^23^, ^24^ In addition, a study suggests that the amount of unsaturated fatty acids in TGs is positively correlated with the expression of cardiac remodeling markers, including left atrial diameter, maximum volume, emptying volume, left ventricular ejection fraction, and LA contractile force.^25^ These studies suggest that lipid metabolism may be involved in the occurrence of AF by altering the structure of the heart.

The specific explanation of the mechanism by which TG functions on cardiac structure and conduction is as follows. A recent basic study has shown that downregulation of adipose triglyceride lipase (ATGL) can promote atrial structure and electrical remodeling, thereby increasing the incidence of AF.^26^ ATGL, a key enzyme, promotes the degradation of TGs in cells and participates in lipid breakdown metabolism,^27^ suggesting the correlation between TG and AF. A mouse model of myocardial cell‐specific ATGL overexpression has shown that in addition to a decrease in TG content in myocardial cells, myocardial cell‐specific ATGL overexpression can also improve energy metabolism, systolic function, and cardiac remodeling in healthy hearts. These studies suggest that interrelated mechanisms underlie AF induced by TG and ATGL.

Since the recurrence rate of AF remains high within 12 months of catheter ablation,^28^, ^29^ continued treatment and regular follow‐up after surgery are required. Our study provides a potential therapeutic target to improve the success rate of ablation. High TG level appears to be a risk factor for recurrent AF, and should be focused in clinical management. Another study also showed a positive correlation between elevated TG level and ischemic stroke in patients with AF.^30^ These data indicate that TG level may be controlled to reduce the risk of AF recurrence after radiofrequency ablation.

## 5 LIMITATION

There are several limitations in the current study. First, patient recruitment in this study started in 2018, and ablation techniques have been upgraded since then, which may have an impact on surgical success.^31^, ^32^ Second, TGs are related to dietary style. Several institutions including the American Heart Association, the European Atherosclerosis Society, and the Danish Society of Clinical Chemistry, now commend nonfasting lipid examination, but all our data were collected from patients fasting before ablation surgery, which may have affected the study results.^4^, ^33^ Third, although we proposed a threshold of TGs, it did not achieve a desired effect. Finally, this study evaluated outcomes over 12 months after ablation, and further studies are needed to assess AF recurrence beyond 12 months.

## 6 CONCLUSION

Our data suggest that an elevated TG level measured before catheter ablation is associated with an increased risk of AF recurrence. Therefore, a good control of plasma TG levels in AF patients before catheter ablation may reduce the risk of postoperative recurrence of AF.

## AUTHOR CONTRIBUTIONS

This manuscript was drafted by Qingqing Gu, Ye Deng, and Jun Wei. Data were statistically analyzed by Qingqing Guand Dabei Cai. Data were statistically analyzed by Tingting Xiao and Yu Wang. The text was polished by Ye Deng and Li Deng. This study was conceived and designed by Ling Sun, Qingjie Wang, and Yuan Ji. All authors reviewed the manuscript.

## CONFLICT OF INTEREST STATEMENT

The authors declare no conflict of interest.

## Supporting information

Supporting information.

## Data Availability

The datasets generated and analyzed in this study are available from the corresponding author on request.

## References

[1] Lippi G , Sanchis‐Gomar F , Cervellin G . Global epidemiology of atrial fibrillation: an increasing epidemic and public health challenge. International Journal of Stroke. 2021;16(2):217‐221. 31955707 10.1177/1747493019897870

[2] January CT , Wann LS , Calkins H , et al. 2019 AHA/ACC/HRS focused update of the 2014 AHA/ACC/HRS Guideline for the Management of Patients With Atrial Fibrillation: a report of the American College of Cardiology/American Heart Association Task Force on Clinical Practice Guidelines and the Heart Rhythm Society in collaboration with the Society of Thoracic Surgeons. Circulation. 2019;140(2):125. 10.1161/CIR.0000000000000665 30686041

[3] Arbelo E , Brugada J , Blomström‐Lundqvist C , et al. Contemporary management of patients undergoing atrial fibrillation ablation: in‐hospital and 1‐year follow‐up findings from the ESC‐EHRA atrial fibrillation ablation long‐term registry. Eur Heart J. 2017;38(17):1303‐1316. 28104790 10.1093/eurheartj/ehw564

[4] Erhard N , Metzner A , Fink T . Late arrhythmia recurrence after atrial fibrillation ablation: incidence, mechanisms and clinical implications. Herzschrittmacherther Elektrophysiol. 2022;33(1):71‐76. 35006336 10.1007/s00399-021-00836-6 PMC8873127

[5] Alonso A , Yin X , Roetker NS , et al. Blood lipids and the incidence of atrial fibrillation: the Multi‐Ethnic study of atherosclerosis and the Framingham Heart Study. J Am Heart Assoc. 2014;3(5):e001211. 25292185 10.1161/JAHA.114.001211 PMC4323837

[6] Boudi FB , Kalayeh N , Movahed MR . High‐density lipoprotein cholesterol (HDL‐C) levels independently correlates with cardiac arrhythmias and atrial fibrillation. J Intensiv Care Med. 2020;35(5):438‐444. 10.1177/0885066618756265 29421988

[7] Huang JY , Liu L , Yu YL , et al. A nonlinear relationship between low‐density‐lipoprotein cholesterol levels and atrial fibrillation among patients with hypertension in China. Ann Palliative Med. 2020;9(5):2953‐2961. 10.21037/apm-20-451 32819119

[8] Guan B , Li X , Xue W , et al. Blood lipid profiles and risk of atrial fibrillation: a systematic review and meta‐analysis of cohort studies. J Clin Lipidol. 2020;14(1):133‐142.e3. 31926850 10.1016/j.jacl.2019.12.002

[9] Shang Y , Chen N , Wang Q , et al. Blood lipid levels and recurrence of atrial fibrillation after radiofrequency catheter ablation: a prospective study. J Interv Card Electrophysiol. 2020;57(2):221‐231. 30955170 10.1007/s10840-019-00543-w

[10] Esan O , Wierzbicki AS . Triglycerides and cardiovascular disease. Curr Opin Cardiol. 2021;36(4):469‐477. 33797418 10.1097/HCO.0000000000000862

[11] Mohammadi‐Shemirani P , Chong M , Narula S , et al. Elevated lipoprotein(a) and risk of atrial fibrillation. J Am Coll Cardiol. 2022;79(16):1579‐1590. 35450575 10.1016/S0735-1097(22)02570-0 PMC9584800

[12] Generoso G , Janovsky CCPS , Bittencourt MS . Triglycerides and triglyceride‐rich lipoproteins in the development and progression of atherosclerosis. Curr Opin Endocrinol Diabetes Obes. 2019;26(2):109‐116. 30694827 10.1097/MED.0000000000000468

[13] Kim SM , Kim JM , Shin DG , Kim JR , Cho KH . Relation of atrial fibrillation (AF) and change of lipoproteins: male patients with AF exhibited severe pro‐inflammatory and pro‐atherogenic properties in lipoproteins. Clin Biochem. 2014;47(10‐11):869‐875. 24201066 10.1016/j.clinbiochem.2013.10.026

[14] Li ZZ , Du X , Guo X , et al. Association between blood lipid profiles and atrial fibrillation: a case‐control study. Med Sci Monit. 2018;24:3903‐3908. 29885277 10.12659/MSM.907580 PMC6024732

[15] Yao Y , Liu F , Wang Y , Liu Z . Lipid levels and risk of new‐onset atrial fibrillation: a systematic review and dose‐response meta‐analysis. Clin Cardiol. 2020;43(9):935‐943. 32720403 10.1002/clc.23430 PMC7462197

[16] Konerman M , Kulkarni K , Toth PP , Jones SR . Evidence of dependence of lipoprotein(a) on triglyceride and high‐density lipoprotein metabolism. J Clin Lipidol. 2012;6(1):27‐32. 22264571 10.1016/j.jacl.2011.08.004

[17] Arnett DK , Blumenthal RS , Albert MA , et al. 2019 ACC/AHA Guideline on the Primary Prevention of Cardiovascular Disease: executive summary: a report of the American college of Cardiology/American Heart Association Task Force on Clinical Practice Guidelines. Circulation. 2019;140(11):e563‐e595. 30879339 10.1161/CIR.0000000000000677 PMC8351755

[18] Nassir F , Bonen DK , Davidson NO . Apolipoprotein(a) synthesis and secretion from hepatoma cells is coupled to triglyceride synthesis and secretion. J Biol Chem. 1998;273(28):17793‐17800. 9651381 10.1074/jbc.273.28.17793

[19] Naji F , Sabovic M . Lipoprotein(a) and inflammation in patients with atrial fibrillation after electrical cardioversion. J Negat Results Biomed. 2011;10:15. 22078666 10.1186/1477-5751-10-15 PMC3229463

[20] Abdelmoneim SS , Rosenberg E , Meykler M , et al. The incidence and natural progression of New‐Onset postoperative atrial fibrillation. JACC Clin Electrophysiol. 2021;7(9):1134‐1144. 33933413 10.1016/j.jacep.2021.02.005

[21] Anselmino M , Ballatore A , Saglietto A , et al. Atrial fibrillation ablation long‐term ESC‐EHRA EORP AFA LT registry: in‐hospital and 1‐year follow‐up findings in Italy. J Cardiovasc Med. 2020;21(10):740‐748. 10.2459/JCM.0000000000000999 32898381

[22] Cohn JS , Lam CWK , Sullivan DR , Hensley WJ . Plasma lipoprotein distribution of apolipoprotein(a) in the fed and fasted states. Atherosclerosis. 1991;90(1):59‐66. 1799398 10.1016/0021-9150(91)90244-w

[23] Shaya GE , Leucker TM , Jones SR , Martin SS , Toth PP . Coronary heart disease risk: low‐density lipoprotein and beyond. Trends Cardiovascul Med. 2022;32(4):181‐194. 10.1016/j.tcm.2021.04.002 33872757

[24] Erqou S , Kaptoge S , Perry PL , et al. Lipoprotein(a) concentration and the risk of coronary heart disease, stroke, and nonvascular mortality. JAMA. 2009;302(4):412‐423. 19622820 10.1001/jama.2009.1063 PMC3272390

[25] Lee HC , Cheng WC , Ma WL , Lin YH , Shin SJ , Lin YH . Association of lipid composition and unsaturated fatty acids of VLDL with atrial remodeling in metabolic syndrome. Sci Rep. 2023;13(1):6575. 37085694 10.1038/s41598-023-33757-0 PMC10121655

[26] Han X , Zhang YL , Zhao YX , Guo SB , Yin WP , Li HH . Adipose triglyceride lipase deficiency aggravates angiotensin II‐induced atrial fibrillation by reducing peroxisome proliferator‐activated receptor α activation in mice. Lab Invest. 2023;103(1):100004. 36748188 10.1016/j.labinv.2022.100004

[27] Cerk IK , Wechselberger L , Oberer M . Adipose triglyceride lipase regulation: an overview. Curr Protein Pept Sci. 2018;19(2):221‐233. 28925902 10.2174/1389203718666170918160110 PMC7613786

[28] Schmidt K , Noureen A , Kronenberg F , Utermann G . Structure, function, and genetics of lipoprotein (a). J Lipid Res. 2016;57(8):1339‐1359. 27074913 10.1194/jlr.R067314 PMC4959873

[29] Tao J , Yang X , Qiu Q , et al. Low lipoprotein(a) concentration is associated with atrial fibrillation: a large retrospective cohort study. Lipids Health Dis. 2022;21(1):119. 36376975 10.1186/s12944-022-01728-5 PMC9661736

[30] Li F , Du X , He L , et al. Relationship between serum lipid levels and ischemic stroke in patients with atrial fibrillation: a nested case‐control study based on the China atrial fibrillation registry. BMC Cardiovasc Disord. 2021;21(1):424. 34496759 10.1186/s12872-021-02237-6 PMC8425053

[31] Kolovou GD , Watts GF , Mikhailidis DP , et al. Postprandial hypertriglyceridaemia revisited in the era of non‐fasting lipid profile testing: a 2019 expert panel statement, main text. Curr Vasc Pharmacol. 2019;17(5):498‐514. 31060488 10.2174/1570161117666190507110519

[32] Keirns BH , Sciarrillo CM , Koemel NA , Emerson SR . Fasting, non‐fasting and postprandial triglycerides for screening cardiometabolic risk. J Nutr Sci. 2021;10:e75. 34589207 10.1017/jns.2021.73 PMC8453457

[33] Buist TJ , Zipes DP , Elvan A . Atrial fibrillation ablation strategies and technologies: past, present, and future. Clin Res Cardiol. 2021;110(6):775‐788. 33089361 10.1007/s00392-020-01751-5

