# Supplementary figures and images for: Plasma triglyceride is associated with the recurrence of atrial fibrillation after radiofrequency catheter ablation: A retrospective study
Source: Clin Cardiol. 2024 May 30;47(6):e24276. doi: 10.1002/clc.24276 (PMC11137329; doi:10.1002/clc.24276)

**Figure S1. Flow diagram of the selection process of patients.**


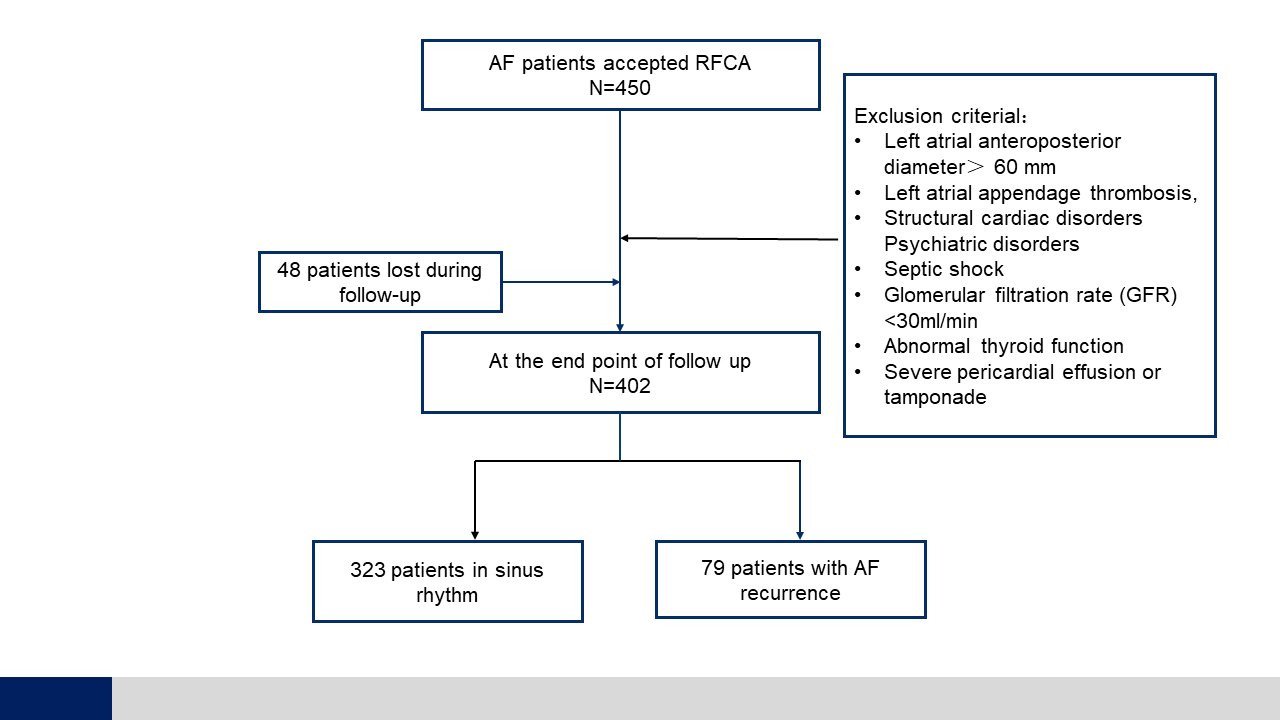

Supplement: Supplementary file 1 — Supporting information. [file CLC-47-e24276-s001.docx]
